# Acute severe autoimmune hepatitis with anti-rods and rings autoantibodies; literature first evidence 

**Published:** 2021

**Authors:** Roberto Assandri, Alessandro Montanelli

**Affiliations:** 1 *Clinical Investigation Laboratory, ASST Crema, Crema, Cremona, Italy*; 2 *Clinical Investigation Laboratory, ASST Bergamo Est, Seriate, Bergamo, Italy*

**Keywords:** Autoimmun hepatitis, Rods and rings antibodies, Autoimmun liver disease

## Abstract

Autoimmune hepatitis (AIH) was defined as a progressive, chronic inflammatory autoimmune liver disease (ALD). The diagnosis of AIH requires the presence of characteristic clinical and laboratory features, and the exclusion of other clinical conditions that cause chronic hepatitis and cirrhosis. AIH can have an acute onset that mimics an acute viral or toxic hepatitis or an acute severe (fulminant, ASF) presentation that satisfies criteria for acute liver. Guidelines from the European Association for the Study of Liver Diseases define ALF with absence of pre-existing liver disease, acute onset of ≤ 26 weeks, coagulopathy (international normalised ratio (INR) ≥ 1.5), and presence of hepatic encephalopathy (HE).

In recent years, autoantibodies (Aab) targeting subcellular structures described as the rods and rings (R&R) pattern in HEp-2 ANA have been presented as a unique and particular case of Aab generation. These R&R structures are composed of inosine monophosphate dehydrogenase type 2 (IMPDH2), and their formation can be induced *in vitro *by several small-molecule inhibitors.

Aab targeting these relatively unknown structures has been observed in Hepatitis C virus (HCV) patients who have undergone treatment with pegylated interferona/ ribavirin (IFN/RBV) therapy.

We presented and characterized a case patient with R&R and SMA Aab in AIH (ASF, fatal, without liver transplantation). To the best of our knowledge, this is the first evidence described in the Literature. Our early experience showed the R&R circulating Aab in one patient with Primary Biliary Cholangitis. This work now demonstrates that R&R Aab can also be present in AIH case.

## Introduction

 Autoimmun hepatitit (AIH) is an autoimmune, progressive, chronic inflammatory liver disorder. 

The diagnosis requires the presence of characteristic clinical and laboratory features strictly linked to the exclusion of other conditions that cause chronic hepatitis and cirrhosis failure (1).

A systematic approach is required to distinguish AIH from other liver diseases with similar clinical features. These include hepatitis associated with viral infections (hepatitis viruses A–E, Epstein–Barr virus (EBV), Cytomegalovirus (CMV) and Herpes simplex virus (HSV)), primary biliary cholangitis (PBC), primary sclerosing cholangitis (PSC), drug-induced liver injury, and Wilson disease (WD) (1). The clinical assessment should furthermore include an evaluation of alcohol consumption. The laboratory assessment should include determination of serum alanine (ALT) or aspartate (AST) aminotransferases, alkaline phosphatase (AP), albumin, IgG, and bilirubin levels. The conventional serologic markers of AIH should also be assessed, including antinuclear antibody (ANA), smooth muscle antibody (SMA), antibody to liver/kidney microsome type 1 (anti-LKM1), and antiliver cytosol type 1 (anti-LC1) (2).

On the basis of circulating autoantibodies pattern, this disease can be classified into two known main types: Type 1 (ANA and SMA) and Type 2 (LKM-1 and/or LC-1) (2). 

AIH could have a broad spectrum of clinical presentations ranging from asymptomatic to fulminant hepatic failure. Guidelines from the American Association for the Study of Liver Diseases define ALF with the absence of pre-existing liver disease, acute onset of ≤ 26 weeks, coagulopathy (international normalised ratio (INR) ≥ 1.5) and presence of hepatic encephalopathy (HE) (3). The following criteria summarized the America guideline: 

1) Acute-AIH – icteric, weeks with no evidence of coagulopathy or encephalopathy

2) AS-AIH – icteric and coagulopathy (INR ≥ 1.5), but no evidence of encephalopathy

3) AS-AIH with ALF – icteric, coagulopathy (INR ≥ 1.5) and encephalopathy (3).

Quite recently, Aab targeting sub-cellular structures described as R&R pattern in ANA HEp-2 screening has been presented as a unique case of Aab generation. These R&R structures are composed of inosine monophosphate dehydrogenase type 2 (IMPDH2),

and their formation can be induced *in vitro *by several small-molecule inhibitors (4). Aab targeting these structures has been observed in hepatitis C virus (HCV) patients who have undergone treatment with pegylated interferon-a/ribavirin (IFN/RBV) therapy (4). The prolonged exposure to interferon-αand ribavirin appeared to increase the prevalence of this Aab (4). In rare cases can anti-R&R Aab be detected in non-hepatitis patients (5).

It is more significant to note that anti-R&R Aab may also be a component of autoimmune responses. However, there has been no report on a clear association of R&R Aab and AD, particularly ALD. Our early experience showed the R&R circulating Aab in one patient with PBC (6). For these reasons, we presented and characterized a case patient with contemporary presence of SMA and R&R Aab in ALF-AIH, without any evidences of HCV and/or other hepatic virus infection. We found no study conducted on this case in the literature. 

## Case report (AIH Type-1 diagnostic work-up)

***Clinical manifestation***

In January 2019, an 86-year-old diabetic woman visited our emergency department with a two-day history of yellowish skin and tea-colored urine. Since a few days before this presentation, she was suffering from nausea, poor appetite and epigastralgia. She denied fever, dyspnoea, habit of alcohol consumption, and history of intravenous drug abuse or liver disease. On admission, her vital signs were as follows: body temperature 36.7°C, blood pressure 128/92 mmHg, pulse rate 74 beats per minutes, and respiration rate 18 breaths per minutes. Her sclera was icteric. The results of her heart and chest examinations were normal. Her abdomen was distended with mild tenderness. 

**Biochemical features**

Baseline Laboratory test results were as follows: serum total bilirubin, 6.83 mg/dL (reference range, 0.2-1.3 mg/dL); alanine aminotransferase (ALT), 100 mU/mL (5-40 mU/mL); aspartate aminotransferase (AST), 118 mU/mL (5-37 mU/mL); alkaline phosphatase, 338 mU/mL (53-141 mU/mL); γ-glutamyltransferase, 629 mU/mL (9-36 mU/mL); lactate dehydrogenase, 381 mU/mL (125-243 mU/mL); albumin, 2.9 g/dL (3.5-5.5 g/dL); and prothrombin time, international normalized ratio (INR) of 1.38 (0.90-1.27). Serum protein electrophoresis revealed elevated polyclonal γ-globulin (gamma fraction 29%, 1.85 g/dL; reference range: 0.8-1.35 g/dL). The serum levels of immunological markers were examined as follows: immunoglobulin G (IgG), 2180 mg/dL (751-1560); and IgA, 410 mg/dL (82-453); and IgM, 293 mg/ dL (46-304). Most relevant serological markers (HBs-Ag, Hbc-Ab, Hbs-Ab, HCV-Ab, HAV IgG, IgM) and other possible serological evidence (CMV IgG/IgM, EBV IgG/IgM) were not found. 

**Immunological profile**

SMA detected by IIF of rodent tissue sections was moderately elevated at a titer of 1:160. 

**Figure 1:**
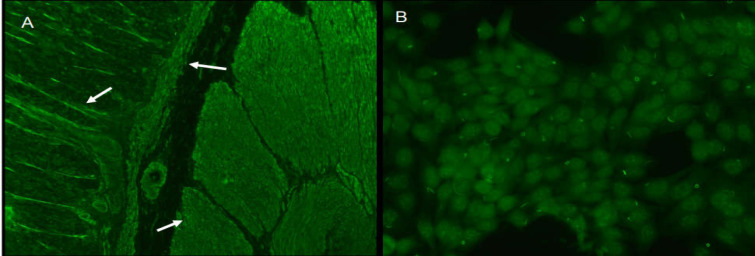
Panel A: SMA detected by IIF of roden tissue sections. Panel B: R&R pattern

IIF on tissue revealed “*tunica muscularis*”, “*lamina muscularis mucosae*” and the interglandular contractile fibrils positivity on the rat stomach at end diluition of 1:160 (Figure 1: Panel A).

**Figure 2:**
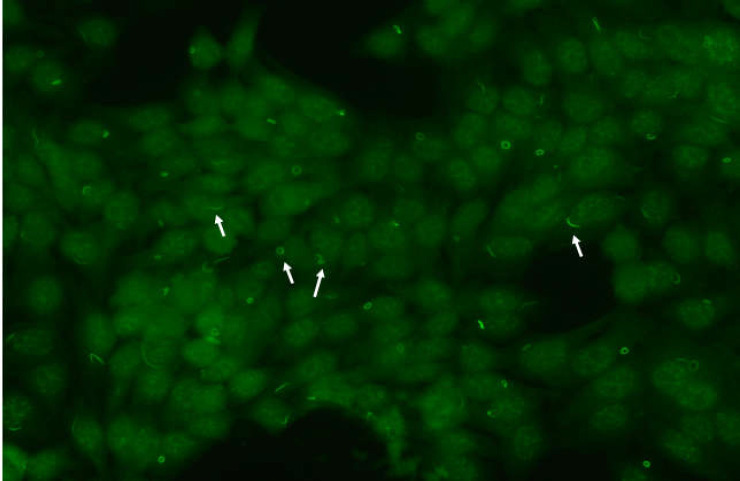
R&R pattern. We observed one to two rods and/or rings per cell including some intermediate structures such as a figure ‘‘8’’, elongated rings, twisted rings, rods with pin loops. Some rods often align adjacent to the nucleus or perpendicular to the nucleus, and rings may be found in the cytoplasm

 Line immunoassay (Euroimmun Lübeck**) **was negative (IgG Antibodies profile: AMA-M2, M2-E3, Sp100, PML, gp210, LKM-1, LC-1 and SLA/LPL).

A distinct cytoplasmic pattern was identified in a routine ANA test using HEp-2 cell slides. ANA were positive at end diluition 1:640 with R&R pattern. The structures recognized by patient serum were distinct cytoplasmic rods and rings (Figure 1, Panel B and Figure 2). We observed one to two rods and/or rings per cell including some intermediate structures such as a figure ‘‘8’’, elongated rings, twisted rings, rods with pin loops. Some rods often align adjacent to the nucleus or perpendicular to the nucleus, and rings may be found in the cytoplasm (Figure 2).

**Hystology**

Histopathological examination of the explanted liver showed massive hepatocellular necrosis with infiltration of numerous inflammatory cells.

**Fulminant liver failure diagnosis**

Abdominal ultrasonography showed parenchymal liver disease with cirrhotic change and ascites. On the basis of the laboratory findings, the patient was diagnosed as having acute form of type 1 AIH on hospital day 6. Despite a5-day intravenous administration of stronger neominophagen C (SNMC) (60 mL/day) and daily transfusion of fresh frozen plasma, her hepatic failure progressed. She developed stage II hepatic encephalopathy, and her serum total bilirubin level peaked at 23.7 mg/dL on hospital day 16. Therefore, she was transferred to our intensive care unit for aggressive treatment. Plasma exchange (PE) therapy of 20 units/day and administration of intravenous hydrocortisone (150 mg/day) were started immediately on hospital. 

The following day, however, her encephalopathy progressed to stage III with an INR of 1.71. Serum bilirubin level from day 16 to day 21 was raised (23.71 mg/dL to 35.1 mg/dL). 

**Figure 3:**
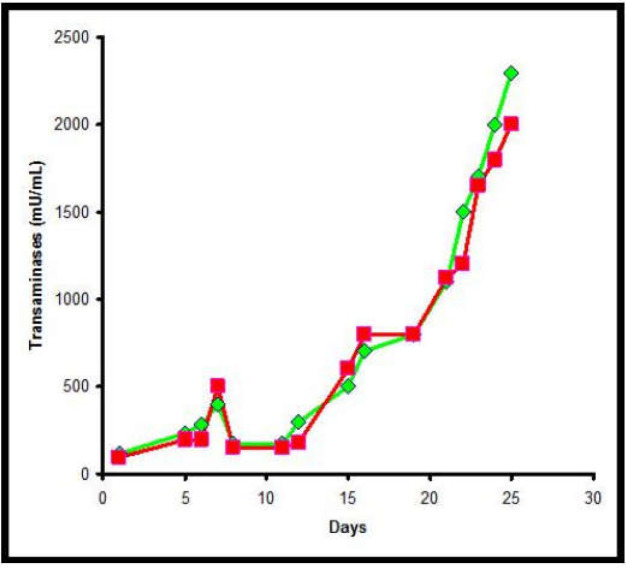
Biochemical parameters. Transaminases trend

The patient further decays until the fatal “*exitus*” at day 25 (Bilirubin level 51mg/dL) after final stage of hepatic encephalopathy. Biochemical findings were reported in Figure 3.

## Discussion

Literature clearly identified the first-line role of laboratory in AIH diagnosis. In fact, on the basis of the pattern of circulating Aab, this disease can be classified into 2 main types: Type 1 (ANA and ASMA) and Type 2 (LKM-1 and LC-1). IIF of rodent tissue section for ASMA/LKM detection and Hep-2 lineage for ANA detection are the hallmarks of AIH diagnosis. 

Our results described above reproduce the methodological approach for a complete characterization of complex antibodies profile. In these situations, it is particularly important that routinely-used techniques are employed in correct combination in order to provide a successful characterization of the fine features of all cases (7-9). However, autoimmune serology remains the Achilles’ heel in the diagnostic work-up for AIH. The study provided a perfect opportunity to discuss the role of traditional laboratory IIF approach. 

As a part of diagnostic protocols, IIF remains the undisputed screening assay in routinely practice. A prominent question is whether the IIF screening test has sufficient sensitivity to detect all clinically relevant Aab. This question has led some laboratories to leave the IFI as a first step of the diagnostic tool because it requires more experience. However, IIF of rodent tissue has been identified by the Committee for Autoimmune serology of the IAIHG as the best technique for the detection of Aab in ALD (2). 

Available commercial substrates have a variable quality. These are treated with fixatives in order to lengthen their shelf life, but this also causes enhanced background staining which can potentially cause difficulties in the interpretation of fluorescence patterns, especially in poorly experienced colleagues. EASL guideline indicates that Aab are considered positive in IIF when present at a dilution titer≥1:40 in adults, while in children the cut-off for positivity is lower, being ≥1:20 for ANA and SMA, and ≥1:10 for anti-LKM1 and LC1 (2). Nevertheless, technique standardization was not unique in all laboratories.

Despite their importance in diagnosis and classification, the pathogenic role of Aab and the mechanisms by which they may potentially contribute to liver damage remain a topic for further research. The target of R&R Aab are conserved intracellular polymeric structures composed of IMPDH2, induced by IMPDH2 inhibitors/glutamine deprivation condition. 

Representing a distinct class of sub-cellular structures, R&R are not associated with any known organelles (10). The R&R pattern is a rare finding of ANA testing in the clinical laboratory, because R&R pattern is related to some HEp2 cell cultures (11). This prevalence is furthermore reported to be between 5% and 20%, especially in the HCV-infected patients cohort (12). 

IMPDH2 is a component of the structures recognized by the R&R Aab (13), and has been related to the HCV treatment with retroviral agent ribavirin, considered as an inhibitor of this enzyme. In vitro studies showed the ability of ribavirin to induce the intracellular structures, which is responsible to an anti- R&R immune response (13). We focused on a hypothetical patient’s cohort/cases series regarding R&R Aab without HCV infection or antiviral agent treatment. Keppeke reported one R&R Aab sample with hepatitis B in a cohort of 57 HCV-R&R positive cases (12). Probst and colleagues demonstrated the presence of R&R Aab in non-HCV patient’s cohort using IMPDH2 as a substrate in a radio-immunoprecipitation assay (11). 

In addition to these evidences, Shaikh’s work reported 39 R&R Aab positive subjects in which only one is HCV-positive patient (14). Literature reported one patient under mycophenolate mofetil (MMF) treatment as an example of inhibitor to IMPDH2 enzymatic activity (15). MMF may cause changes in the IMPDH2 molecular structure as ribavirin. Climent reported eight patients with R&R Aab and concomitant AD, and it was hypothesized that these Aab may be a result of autoimmune responses (16). The same authors performed liver biopsy in 54 R&R- positive patients. These biopsies revealed a high degree of hepatic fibrosis in 38 patients, but they didn’t have specific histological pattern of PBC, AIH and other overlapping conditions (16).

Our work demonstrated that R&R Aab can be present in PBC (6) and now in AIH case. So, we establish that R&R Aab reactivity is strictly connected with hepatic diseases, including ALD. When does the R&R immune response in AIH patient develop? What molecular structure can mimic IMPDH2? In patients with increased genetic susceptibility to AIH, immune responses to liver antigens could be triggered by molecular mimicry (17). The hypothesis that exposure to self-mimicking exogenous sequences can trigger AIH is supported by a case report in a child who acquired HCV infection after liver transplantation for end-stage liver disease due to α1antitrypsin deficiency (18). These data suggest that HCV infection initiated an antiLKM1 immune response and support the involvement of molecular mimicry in the pathogenesis of AIH (18).

An interesting animal model characterized by deletion of medullary thymic epithelial cells which regulate T cell tolerance, shows that the mice do not have multi-organ autoimmune disease, as might be expected. Instead, the animals develop a condition closely resembling human AIH1, production of ANA, anti-SLA antibodies and antibodies directed to liver-specific antigens, supporting a key role of regulatory mechanisms in the pathogenesis of AIH (19). Molecular mimicry, loss of self-tolerance, and self-antigen presentation driven by reduction of T_Reg_-cell prove clinical manifestations in AIH (18).

In a very recent experiment, Chang et al. investigated IMPDH2 filament formation in human and murine T cells. Authors discovered extensive in vivo IMPDH filament formation in mature T cells, B cells, and other proliferating splenocytes of normal, adult Black 6 (B6) mice (20). IMPDH2 are considered a DNA-binding trascritpional repressor and may induce reduction of T_Reg_-cell. In an over-expressed condition (as AIH), loss of self-tolerance could induce a response against IMPDH2, showing a epiphenomenon R&R pattern. Particularly, imbalance of effector T cells-receptor and regulatory T cells, and the consequent direct action over B lymphocytes are possible mechanisms leading to autoimmune manifestations (20). This paper remarks the association of R&R Aab to ALD. To solve this issue, other studies had needed. Literature review indicated that the R&R pattern cannot be considered only a side effect of the antiviral treatment. In a very recent paper Lei Zhang and co-workers retrospectively investigated the clinical significance of R&R Aab in ANA test samples of Western China. They concluded that R&R Aab have a low prevalence, and there is no gender difference. R&R Aab antibodies existed other diseases besides hepatitis C, such as HBV, some autoimmune diseases and renal insufficiency (21). Our case should be considered a touchable example of this situation: R&R Aab in patients without any clinical/laboratory signs or symptoms of viral Hepatitis or pharmacological therapy. We clearly demonstrated that R&R Aab are not exclusively hallmark of HCV- ribavirin treated patients, but the expression of a complex, partial unknown and hypothetical molecular pathway.

## Conflict of interests

The authors declare that they have no conflict of interest.
